# Extensive Intestinal Resection Triggers Behavioral Adaptation, Intestinal Remodeling and Microbiota Transition in Short Bowel Syndrome

**DOI:** 10.3390/microorganisms4010016

**Published:** 2016-03-08

**Authors:** Camille Mayeur, Laura Gillard, Johanne Le Beyec, André Bado, Francisca Joly, Muriel Thomas

**Affiliations:** 1 Micalis Institute, INRA, AgroParisTech, Université Paris-Saclay, 78350 Jouy-en-Josas, France; camille.mayeur@jouy.inra.fr; 2 Inserm UMR 1149, UFR de Médecine Paris Diderot, Université Paris Diderot, Sorbonne Paris Cité, DHU Unit, APHP, F-75890 Paris, France; gillard.laura@yahoo.com (L.G.); johanne.le-beyec@inserm.fr (J.L.B.); johanne.le-beyec@inserm.fr (A.B.); francisca.joly@aphp.fr (F.J.); 3 Université Pierre et Marie Curie and Department of Endocrine and Oncologic Biochemistry, APHP La Pitié-Salpêtrière Hospital,73013 Paris, France; 4 Department of Gastroenterology and Nutrition Support, APHP Beaujon Hospital, 92110 Clichy, France

**Keywords:** mal-absorption, dysbiosis, colon, surgery, lactate

## Abstract

Extensive resection of small bowel often leads to short bowel syndrome (SBS). SBS patients develop clinical mal-absorption and dehydration relative to the reduction of absorptive area, acceleration of gastrointestinal transit time and modifications of the gastrointestinal intra-luminal environment. As a consequence of severe mal-absorption, patients require parenteral nutrition (PN). In adults, the overall adaptation following intestinal resection includes spontaneous and complex compensatory processes such as hyperphagia, mucosal remodeling of the remaining part of the intestine and major modifications of the microbiota. SBS patients, with colon in continuity, harbor a specific fecal microbiota that we called “lactobiota” because it is enriched in the *Lactobacillus/Leuconostoc* group and depleted in anaerobic micro-organisms (especially *Clostridium* and *Bacteroides*). In some patients, the lactobiota-driven fermentative activities lead to an accumulation of fecal d/l-lactates and an increased risk of d-encephalopathy. Better knowledge of clinical parameters and lactobiota characteristics has made it possible to stratify patients and define group at risk for d-encephalopathy crises.

## 1. Introduction

Short bowel syndrome (SBS) is a well-known cause of intestinal failure (IF) [1]. SBS occurs in patients who have an extensive resection of the small bowel (RSB) that leaves less than 150/200 cm. Part or the entire colon may have also been removed. These patients suffer from severe water and nutrient mal-absorption, so they often receive nutritional support by parenteral nutrition (PN) to supplement their oral intake [2,3,4].

A complete understanding of the physiopathology of SBS and post-surgery adaptations may reveal the spontaneous processes that compensate for the reduction of the absorptive surface. A better knowledge of these adaptive mechanisms may allow us to rationally manage patient nutrition, to reduce the need for PN and to prevent d-encephalopathy crisis. This review focuses on the overall adaptations described for adult SBS patients, and does not cover this pathology in children.

Adaptation following intestinal resection includes spontaneous and complex compensatory processes such as hyperphagia and intestinal remodeling of the remaining part of intestine. In SBS adults, hyperphagia is reflected by an increase in nutritional intake after surgery. Adaptations of the intestine triggered by surgery must take into account adaptations of both the mucosa and the microbiota. Indeed, gut function results from an intricate relationship between eukaryote and prokaryote partners. The intestinal microbiota influences a variety of intestinal functions, including the development and maturation of the mucosal epithelium and immune system [5,6,7,8,9]. The phylogenetic core of healthy adult human microbiota includes *Firmicutes*, *Bacteroidetes* and *Actinobacteria* [10,11]. The composition and metabolic functions of intestinal microbiota are mostly altered by age, nutrition, environment and health status [12,13,14]. We mainly report the post-surgery adaptations occuring in the remnant colon, because preservation of at least a part of the colon is a determining factor in reducing the need for PN and contributes to the clinical outcomes of SBS in adults. In the context of increasing knowledge on the role of microbiota, we emphasize our results describing the activities and composition of microbiota after surgery in SBS adults. We also propose that d-lactate accumulation in feces may be associated with a higher risk of metabolic acidosis and d-encephalopathy. 

Intestinal surgeries that result in shortening of the intestinal tract are increasingly proposed for the treatment of morbid obesity or type II diabetes [15]. Studies of the physiopathology of SBS may reveal mechanistic clues for the adaptive process following the loss, or the exclusion, of a part of intestine. 

## 2. Intestinal Failure and Short Bowel Syndrome

Intestinal failure (IF) occurs in various gastrointestinal diseases such as gut motility disorders, mechanical obstruction, intestinal fistula, extensive small bowel mucosal disease, volvulus or systemic conditions such as mesenteric infarction, and post-radiation enteritis requiring an extensive small bowel resection. IF is defined as a reduction of gut function below that minimally necessary for the absorption of macronutrients and/or water and electrolytes, such that intravenous (IV) supplementation is required to maintain health and/or growth [1]. Three different types of IF are reported based on duration : (i) acute, short-term and usually self-limiting conditions; (ii) prolonged acute conditions, often in metabolically unstable patients, requiring complex multi-disciplinary care and IV supplementation over long periods; and (iii) chronic reversible or irreversible conditions, in metabolically stable patients, requiring intravenous supplementation over a long period. In adults, massive intestinal resection leaving less than 200 cm of small bowel defines SBS, and a small bowel length of <100 cm is highly predictive of permanent IF [16,17]. SBS is one of the main causes of IF, but bariatric surgery can also lead to IF [18].

While the true prevalence of SBS in adults is unknown, it is estimated to be 1.4 cases per million people in Europe. It varies by region, from 0.4 per million in Poland to approximately 30 per million in Denmark [19]. The prevalence of SBS is lower in regions that lack major intestinal rehabilitation centers and efficient home PN or IV programs, likely because of under-reporting and the inability to adequately treat these patients. Nonetheless, this patient population is growing: a leading intestinal rehabilitation center in Denmark reported a >2-fold increase in the number of patients with PN or IV-dependent SBS per decade during the past 40 years [20].

The symptoms and severity of SBS are linked to (i) anatomic sections of the resected intestine remaining; (ii) the length and absorptive capacity of the remnant bowel and (iii) the cause of primary disease. Resection of the small bowel results in three different anatomic anastomoses: (i) enterostomy (≤40–80 cm RSB, in 17% of patients); (ii) jejuno-colonic (≤40–80 cm RSB, in 68% of patients); and (iii) jejuno-ileo-colonic (≤20–80 cm RSB, in 15% of patients) [1]. SBS patients develop clinically significant mal-absorption and dehydration with electrolyte disturbances due to the reduced absorptive area, accelerated gastrointestinal transit time and modification of the gastrointestinal intraluminal environment. As a result, SBS patients require PN [1]. Adaptive changes following resection explain why some patients can be weaned off PN. The degree of intestinal adaptation depends on the underlying pathology that required resection, the anatomic sections of the intestine that remain and the length of remaining bowel [17,21]. The majority of intestinal adaptation in adults is thought to occur during the first 1–2 years following resection, but no objective, clinically practical markers have established the time course or extent of adaptation in humans [22]. Preservation of the colon is a determining factor in reducing the need for PN in SBS patients [17,23]. The probability of PN independence is 47% 5 years after surgery and is significantly associated with a remnant small bowel length greater than 75 cm, a large part of remaining colon and a postoperative citrulline concentration greater than 20 µmol/L [24].

## 3. Endocrine Functions in Adult SBS

Intestinal absorption is inversely correlated with transit time and depends on the intestinal resection site in SBS patients [25,26]. The accelerated transit time observed in jejunostomy and ileum-resected SBS patients favors nutrient mal-absorption because the precise control of the transit time is required to maintain equilibrium of hydro-electric and energetic balances [25,27]. Functional adaptation of the intestinal endocrine cells has been observed in SBS patients. The fasting plasma levels of GLP1 and GLP2 were elevated in extensive gut resection patients with preserved colon and increased further in response to breakfast [28]. These two hormones are produced by the enteroendocrine L cells located in the ileum and colon. GLP2 increases the absorptive surface via its trophic action on mucosal epithelial cells and GLP1 slows gastric emptying and intestinal transit [29,30,31,32]. Both of these actions (increased time and surface of nutrients contact) may improve SBS gut absorption [30,33]. The therapeutic suppression of motility may help to restore gut function. GLP2 treatment induces the increased morphological adaptation of the remnant intestine in both humans and animal models [34,35,36]. The administration of teduglutide, a gut-specific GLP-2 analogue, permitted a >20% reduction in the intravenous requirements of over 63% of patients after 6 months of treatment in controlled clinical trials [37]. Teduglutide significantly decreased stool wet weight and fecal energy excretion [38]. It also significantly increased villus height, crypt depth and the mitotic index in jejunum from SBS patients with end jejunostomy, whereas crypt depth and the mitotic index did not change in colonic biopsies from SBS patients with an intact colon [38]. These novel approaches using GLP-2 analogs have aimed to enhance the natural adaptation process, and have reduced the intravenous caloric needs by approximately 500 kcal/day. While some patients have been weaned off of PN, more have at least been able to reduce the frequency of infusion [37]. Patients who received teduglutide showed significant increases in plasma citrulline (a marker reflecting enterocyte mass) compared to patients receiving placebo in 2 phase III studies [39].

## 4. Hyperphagia in Adult SBS

Dietary intervention is essential to improve the quality of life of SBS patients. For example, continuous tube feeding (exclusively or in conjunction with oral feeding) following the postoperative period significantly increases net absorption of lipids, proteins, and energy compared to oral feeding [2]. Hyperphagia is reported in 70% of adult SBS patients and is defined by oral intake >1.5 times their resting energy expenditure (REE) [40]. In the first cohort that we studied, SBS patients (*n* = 12, jejuno-colonic anastomosis) had a total oral intake of 2600 kcal/day, equivalent to 2.0 times their REE [41]. In the second cohort, composed of 16 SBS patients (jejuno-colonic anastomosis), 11 had an intake/REE ratio greater than 1.5 [40]. Hyperphagia may be an important mechanism to compensate for the reduction of intestinal length, improve patient survival and reduce the need for PN [42]. The balance between orexigenic and anorexigenic hormones is not known in SBS; the mechanisms that control hyperphagia are not well understood. One study has suggested that the fasting postprandial levels of ghrelin and PYY may be altered in SBS patients but neither ghrelin nor PYY levels were significantly related to energy intake or absorption [43]. As hyperphagia leads to an increase of the quantity of nutrients that pass into the gastrointestinal tract, this adaptive mechanism may indirectly contribute to the structural and functional adaptations of the mucosa observed in the remaining gut [42,44].

## 5. Morphological Adaptation of Colon Mucosa in Adult SBS

**Figure 1:**
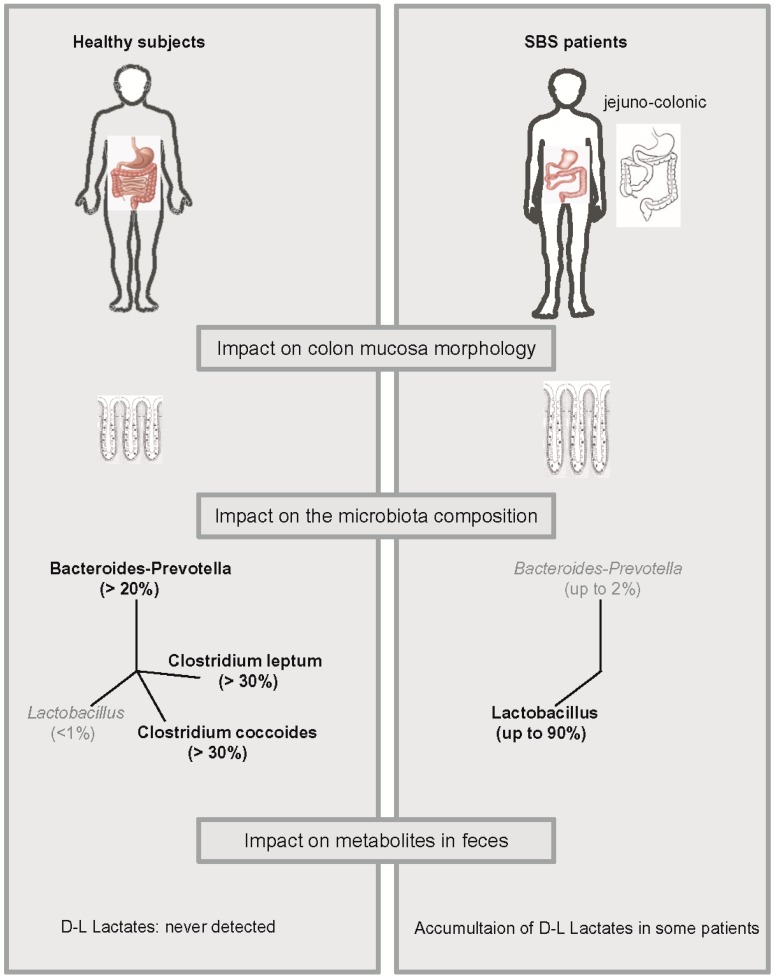
Intestinal resection induces a modification in colon mucosa morphology, microbiota composition, and lactate accumulation in feces.

We observed adaptive changes in colon mucosa including increased crypt depth and increased numbers of colonocytes per crypt without modification of the proliferation/apoptotic ratio (Figure 1). In this study, hyperphagic adult SBS patients (*n* = 12) with a jejuno-colonic anastomosis were examined at least 2 years after the last surgery and were compared with 11 healthy controls (Figure 1) [41]. This was the first study that showed controlled non-pharmacological hyperplasia in the colon of patients with SBS. An increase in villus height, crypt depth and proliferative index has also been described in animals with SBS [45]. No gut epithelium restructuring was observed in resected rats fed exclusively by PN without oral intake [46], suggesting that this structural intestinal adaptation depends on the presence of luminal nutrients. Indeed, the absence of nutrients in the lumen led to mucosal atrophy that was reversed by oral intake [47]. In addition to their ability to trigger morphological adaptation, luminal nutrients also led to functional adaptation of the mucosa and improved absorption [2].

## 6. Transporter Adaptation in Adult SBS

Functional absorptive adaptation of the gut has also been reported in SBS patients via the induction of glucose absorption by the intestinal mucosa, net protein intake [44,48] and calcium absorption [49]. We have found that the Na^+^/H^+^ exchanger (NHE2 and NHE3) and H^+^-coupled oligopeptide (PepT1) transporter mRNA levels in colon were unchanged compared to controls both in humans and rodent models [41,50]. In contrast, increased PepT1 and NHE2/NHE3 mRNA levels have been reported in the colon of SBS patients [51] and in rodents [52]. This apparent discrepancy may be due to the different nutritional status of patients, the use of different animal models and different times between the surgery and the study. In a rat model, glucose transporter (SGLT1) mRNA, protein and activity levels increased in the small bowel remnant [53,54]. An increase in aquaporin 1,3, 7 and 8 gene expression in the small bowel remnant has also been reported [55].

It is difficult to conclude whether, which and where precisely in the gut transporters are modified/enhanced after resection. Nonetheless, published studies suggest that the expression of some transporters may exhibit adaptive flexibility that leads to their over-expression under permissive conditions depending on the type of surgery and the nutritional supply.

## 7. Colon Microbiota Composition and Its Metabolic Functions in Adult SBS

The human gastrointestinal tract is colonized by a dense, complex community of microorganisms, largely composed of anaerobic bacteria in healthy colon. This gut microbiota exerts numerous physiological functions that are important for host homeostasis. The predominant bacterial community is host-specific and relatively stable over time in healthy adults [56]. Gram positive bacteria (*Firmicutes*) predominate, followed by the *Bacteroides* group. The healthy human fecal microbiota is mainly composed of a phylogenenic core containing *Firmicutes*, *Bacteroidetes*, and *Actinobacteria* [11].

Bacteriological analysis, based on culture-dependent methods, shows that *Lactobacilli* dominated the microbiota in SBS patients [57]. The composition and metabolic functions of the gut microbiota in SBS patients were compared to those of healthy controls by using molecular methods to better understand the role of the gut microbiota on the pathophysiology of SBS [40,58]. Overall bacterial diversity is reduced in SBS patients [58] and animal SBS models [59]. Although the overall amount of bacteria in patients and controls were not quantitatively different, the composition of fecal and colon mucosa microbiota was unbalanced in SBS: *Lactobacillus* dominated and anaerobic bacteria (*C. leptum*, *C. coccoides* and *Bacteroides*) were under-represented [40,57,58]. The overload in *Lactobacillus* should be considered massive, since this group contributes little (<1%) to the complex microbiota population in healthy humans (Figure 1). Moreover, some strains like *Lactobacillus mucosae* are found in SBS patients while never having been described in healthy adults. Therefore, SBS-related microbiota is enriched for *Lactobacillus* and depleted of oxygen sensitive bacteria. This mixture is called a lactobiota [40] (Figure 1).

The essential role of the colon in SBS patients is linked to its own absorptive capacity, the metabolic capacity of its specific lactobiota and the reciprocal cross-talk between the lactobiota and colon mucosa. The metabolic functions of human gut microbiota depend on the nature and quantity of substrates available for fermentation in the colon. The origin of theses substrates are both exogenous (dietary nutrients) and endogenous (produced by the host) [60]. After resection, the substrates that arrive in the colon are abundant and poorly digested. In healthy individuals, the fermentation of substrates by gut bacteria helps to maintain gut ecosystem diversity and to recover energy from nutrients saving up to 180 Kcal/day [61]. More than 1000 Kcal/d are saved in SBS patients [62,63,64,65]. The bioconversion of macromolecules by the gut microbiota into metabolites is carried out by bacteria of various functional groups (sharing similar and complementary activities) resulting in metabolic trophic chains and homeostasis with colon epithelium. In SBS, the trophic chains and the fermentative end-products are produced by lactobiota and are different from those produced by healthy microbiota. Diet and anatomical specificity of the gut are major driving forces that shape the microbiota [66]. In our patients, the partition between the three macronutrients (proteins, carbohydrates and lipids) is quite similar to their healthy counterparts [40,58]. Thus, the difference in gut microbiota is more likely to be related to physical and chemical luminal constraints than to qualitative differences in diet between patients and controls. The resection leads to deep lumen alterations favorable to lactobiota. In SBS patients, it is possible that the level of oxygen may be too high to favor the growth of anaerobic bacteria because of the short length of remnant small intestine and colon. In addition to the potential role of O_2_, the low fecal pH of SBS patients, rapid transit time, disruption of enterohepatic circulation and large amount of undigested nutrients arriving at the remaining colon may modify the luminal environment. This may favor the proliferation of lactic-acid-producing bacteria at the expense of that of aero-sensitive bacteria.

## 8. Fecal d-and l-Lactate and Clinical Risk for d-Encephalopathy in Adult SBS with Colon in Continuity

d-lactic acidosis normally occurs in ruminants, and only infrequently in humans [67]. This rare form of lactic acidosis is seen mostly in SBS patients who have a part or an intact colon in continuity [68]. Some cases have also been described in patients with severe diabetic ketoacidosis or after the ingestion of toxic chemicals such as propylene glycol. The principle agent responsible for metabolic acidosis is d-lactic acid, even if the mechanisms involved in its toxicity are not well understood. d-lactic acid predominantly affects the central nervous system. However, other organic acids produced in the colon may also be potentially neurotoxic. These include mercaptans, aldehydes, amines, and alcohols, which can potentially act as false neurotransmitters and give rise to clinical symptoms [69]. Neurotoxicity may also be due to metabolic disturbances. d-lactate is converted to pyruvate. The cerebellum is a potential target for damage in d-lactic acidosis. This is because it has a limited supply of pyruvate dehydrogenase, the enzyme required to convert pyruvate to acetyl co-A. Indeed, the levels of pyruvate dehydrogenase in the cerebellum are not sufficient to metabolize all of the additional pyruvate and this, in combination with thiamine deficiency, may result in neurologic symptoms [70].

The symptoms of d-lactate acidosis are often transient making it difficult to diagnose. Symptoms may include slurred speech, ataxia, altered mental status, gait disturbance, weakness, aggressive behavior, explosive speech, feeling drunk, psychosis, or even coma [70]. A high index of clinical suspicion is key in diagnosing d-lactate acidosis in patients with elevated anion gap metabolic acidosis, normal serum l-lactate levels, a history of SBS, and the above-described clinical features. In this context, the clinical history of the patient should be carefully considered. Patients often present a history of prior neurological symptoms following consumption of a meal rich in carbohydrates. Early identification and correction of metabolic abnormalities result in an improvement in the neurological symptoms. Treatment depends on the clinical status of the patient. The treatment plan requires the removal of the offending agent (carbohydrates, propylene glycol, or exogenous d-lactate) and treatment to decrease the level of d-lactate-producing bacteria in the colon. Poorly absorbed oral antibiotics are most effective and include: clindamycin, vancomycin, neomycin, and kanamycin. Strategies for preventing future occurrences must be implemented once the acute phase is under control. Long term management should focus on avoiding the substrates responsible for d-lactate production. Simple carbohydrate restriction may be useful as they are metabolized to d-lactate more rapidly. Recently, a child with SBS and recurrent, debilitating d-lactic acidosis was successfully treated with fecal transplantation [71]. 

The manner in which d-lactate exerts its clinical influence is complex and there are multiple factors that contribute to the outcomes. Moreover, a survey of SBS patients is difficult since measurement of serum d-lactate concentration is not routinely done and the results come back after the d-lactic acidosis crisis. Understanding the pathophysiological mechanisms for the effects of d-lactate should help physicians to identify d-lactate acidosis to improve preventive and therapeutic strategies [70]. 

Lactate is produced both by eukaryotic and prokaryotic cells and does not accumulate in healthy human feces because it is absorbed by intestinal cells or used by lactate consuming bacteria [72,73,74]. In a cohort of 16 hyperphagic adult SBS patients with a jejuno-colic anastomosis, we observed that the feces of 56% SBS patients contained d and l-lactates, while that of 44% did not (Figure 1). We propose the classification of SBS patients into two subtypes: those with no detectable lactate in their feces (NLA subgroup), and those with d- and l-lactate in their feces (LA subgroup). The LA subgroup contains patients at risk for encephalopathy while no patients from the NLA subgroup developed D-acidosis.

l-lactate is rapidly metabolized by the l-isomer-specific lactate dehydrogenase, whereas the d-lactate enantiomer is toxic for neurons independently of acidosis [75]. Each *Lactobacillus* species identified in SBS lactobiota was characterized based on their specific *in vitro* d and l-lactate production profile. In addition to their equivalent capacity to synthesize both lactate enantiomers, Lactobacilli also possess the dl-lactate racemase resulting in the production of either one or both d and l lactate. The total amount of fecal lactates and the fecal d/l lactate ratio were different and specific for each individual of the LA sub-group, and up to now it has been difficult to associate a specific profile of lactobiota with a greater risk of D-acidosis [40]. It could be interesting to study (by sequencing) the lactate-producing *vs.* lactate-consuming strains in lactobiota of SBS patients. We also observe that the d/l faecal lactate ratio is a most relevant index for d-encephalopathy risk rather than the total d- and L-lactate amount. To increase the number of patients and cases, we encourage monitoring of the d/l-fecal lactates, when patients are suspected to be at risk. In addition, there was an inverse correlation between plasma HCO3^−^ and fecal lactate levels. Two patients in the LA subgroup had a fecal d/l lactate ratio of greater than two and were at risk of d-encephalopathy. We thus consider the plasma HCO3^−^ value and the d/l fecal lactate ratio to be relevant risk markers for d-encephalopathy [40]. Fecal samples are routinely analyzed for d and l-lactate content in a French coprology laboratory in Paris (Pité-salpêtrière hospital) to aid clinicians in their medical diagnoses. 

## 9. Conclusions

**Figure 2:**
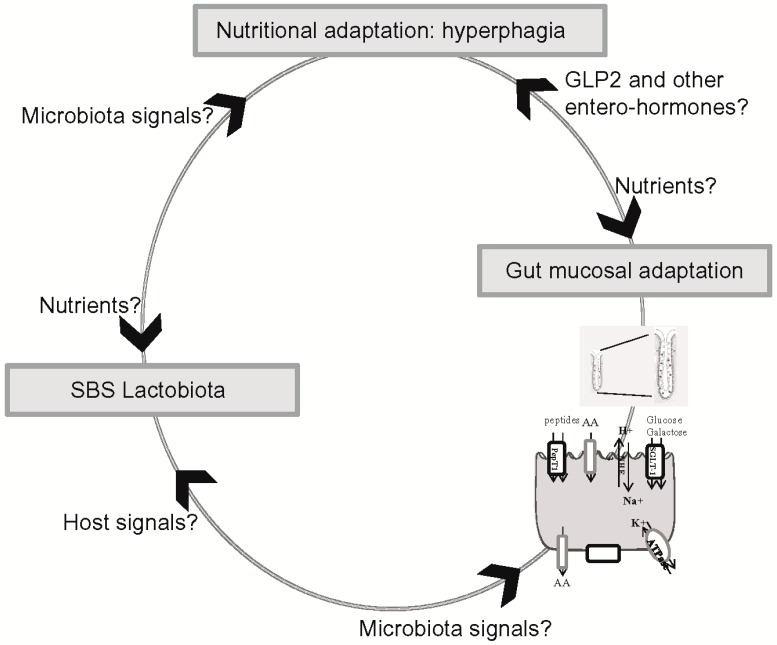
What are the signals involved in the control of food intake, modification of the intestinal mucosa and the lactobiota in SBS?

The morphological and functional alterations that have been described in SBS may contribute to improving nutrient and fluid absorption in the remnant bowel. Improved comprehension of the cellular, molecular and microbiological mechanisms involved in the functional adaptation of the remnant bowel in SBS could help clinicians to optimize overall nutritional absorption, thus reducing or weaning patients off PN and preventing d-encephalopathy crisis (Figure 2). It would now be informative to make molecular and functional links between the three levels of signal integration: the control of food intake, remodeling of the intestinal mucosa and balancing of the microbiota. Important issues to address in the future are (1) the nutritional peripheral hormones and central-hypothalamic neuropeptides that control food intake in SBS patients; (2) whether the mucosal adaptation of the remnant gut is involved in hyperphagia in SBS patients; and (3) whether the SBS lactobiota contributes to hyperphagia and mucosal hyperplasia.
